# Synthesis and catalytic performance of polydopamine supported metal nanoparticles

**DOI:** 10.1038/s41598-020-67458-9

**Published:** 2020-06-26

**Authors:** Haoqi Li, Jiaxin Xi, Adrienne G. Donaghue, Jong Keum, Yao Zhao, Ke An, Erica R. McKenzie, Fei Ren

**Affiliations:** 1 Department of Mechanical Engineering, Temple University, Philadelphia, PA 19122 USA; 2 Department of Civil and Environmental Engineering, Temple University, Philadelphia, PA 19122 USA; 3 Chemical and Engineering Materials Division, Oak Ridge National Laboratory, Oak Ridge, TN 37830 USA; 4 Center for Nanophase Materials Sciences, Oak Ridge National Laboratory, Oak Ridge, TN 37830 USA

**Keywords:** Nanoparticles, Synthesis and processing

## Abstract

Polydopamine (PDA) is an emerging nature-inspired biopolymer material that possesses many interesting properties including self-assembly and universal adhesion. PDA is also able to form coordination bonds with various metal ions, which can be reduced to metal nanoparticles (NPs) as a result of thermal annealing under protective environment. In this study, PDA has been utilized as a support material to synthesize Pt NPs in an aqueous solution at room temperature. The catalytic performance of the resulting PDA-Pt nanocomposite was evaluated using an electrochemical workstation which showed comparable activity to Pt/C material for hydrogen evolution reaction (HER). Furthermore, Cu, Ni, and Cu–Ni NPs supported on PDA were also obtained using this strategy with assistance of subsequent thermal annealing. The phase evolution of the NPs was studied by in-situ X-ray diffraction while the morphology of the nanoparticles was investigated using electron microscopic techniques. Preliminary results showed the NPs supported on PDA also possessed HER activity. This work demonstrates that PDA can be utilized as a potential support for synthesis of metal NPs that can be exploited in engineering applications such as catalysts.

## Introduction

With over a decade of research and exploration, polydopamine (PDA) has been found to possess multiple interesting properties such as capabilities of universal coating and self-assembly in alkaline solutions^1^. Thanks to its catechol functional groups, PDA can form coordination bonding with metal ions, especially with transition metal ions^2^. A handful of studies have reported making metal-PDA (M-PDA) composites from PDA materials containing Fe^3+^, Ag^+^, Au^3+^, Pd^2+^, Co^2+^, Cu^2+^, Ni^2+^, W^6+^, and etc.^3–8^. Direct reduction of noble metals such as Ag, Au, and Pd have been demonstrated without the addition of other reductants^5, 9, 10^. Although non-precious metal ions cannot be reduced by PDA alone, other methods were applied. Heat treatment was usually used in these processes to convert metal ions to metals or metal oxides. On the other hand, electron beam reduction employed in our previous work highlighted a potential method for in-situ metal ion reduction^11^.

Carbonization of PDA in inert atmosphere can convert insulating PDA into electrically conductive cPDA^12^. The highest electrical conductivity reported^13^ is 2.6 × 10^5^ S/m and this conductivity is similar to or even higher than reduced graphene oxides. Li et al. ^14^ have successfully fabricated wafer-scale cPDA transferred onto a soft substrate which could be potentially used to make flexible electronic circuits and sensors. In addition, our previous research^15^ found that annealing the metal-doped PDA not only converted PDA into conductive cPDA, but also converted metal-ions into nanoparticles (NPs). One potential application of such supported metal NPs is catalyst for chemical reactions, such as water splitting.

Electrochemical water splitting has attracted much attention for hydrogen production owing to its abilities to produce high-purity hydrogen which can be used as a renewable energy storage media with zero carbon emission when consumed. The best performing single-phase element catalyst for hydrogen evolution reaction (HER) is Pt, which is not economically attractive for commercialization. Researchers have been focused on improving the overall efficiency of catalysis while reducing cost by employing methods such as reducing the size or dimension of the materials^16^, attaching catalysis to porous substrates, alloying Pt with non-precious metals^17^, and replacing Pt with non-precious metal alloys^18^. While many studies have focused on the catalytic performance characterization (i.e. their over-potential and degradation), optimization for the commonly used fabrication method, which employs Nafion™ as a conductive binder to bind metal NPs onto conductive substrates, still demands improvements.

In search of new support materials for active nanoparticles, we utilized PDA’s universal adhesive property and its chemical versatility to form coordination bonds with metal cations to synthesize self-supported metal NPs catalysis for HER. Literature studies showed that carbonized PDA exhibited good catalytic performance in HER and oxygen evolution reaction likely due to its high surface area and abundant exposed active sites resulting from its porous structure^19, 20^. Another study showed PDA coating also helped to protect the metal NPs supported on the carbon nanohorn (CNH) and increased their stability^21^. In this work, Pt NPs with uniform distribution were directly reduced onto the conductive substrate. HER performance was compared against commercially available Pt/C powder mounted onto glassy carbon electrodes. Results illustrate our novel processing method as a simplified approach without sacrificing the performance. The new processing method was further extended to synthesize Cu, Ni, and Cu-Ni alloy NPs in thin films and powders of M-PDA. Their morphology and structure were studied, and their HER performance was also measured and discussed.

## Experimental methods

### Synthesis of metal NPs on PDA materials

To make Pt samples, PDA coating on graphite rods (99.9995%, Alfa Aesar, Haverhill, MA) was first synthesized and washed. PtCl_4_ powder (> 99.9%, Sigma-Aldrich, St. Louis, MO) was dissolved in deionized (DI) water. The coated graphite rod was then immersed in 50 mM PtCl_4_ solution for 3 h. The PDA coating would directly reduce the Pt^4+^ ions into Pt NPs, which remained on the PDA coating. The product was then heat treated at 800 °C using the same procedure as previously described [12].

Cu, Ni, and bi-elemental alloy attached to PDA were synthesized in two steps. Step one consisted of adding 1:1 molar ratio of metal chlorides (Sigma-Aldrich, St. Louis, MO) and dopamine hydrochloride (99%, Alfa Aesar, Haverhill, MA) into 50 mL of 50 mM TRIS buffer (pH adjusted to 8.5, Thermo Fisher Scientific Inc., Waltham, MA). Substrates to be coated were submerged into the solution. Typical synthesis process would last for 24 h. Powder samples were collected by centrifugation. Both coated substrates and powder were rinsed three times and then dried in an air oven at 50 °C overnight. In step two, dried coatings and powders were heat treated under N_2_ environment at 600 °C, 800 °C, 1,000 °C for 1 h in a tube furnace.

### Structural and chemical characterization

Scanning electron microscopy (SEM, 10 kV, FEI Quanta450 FEG SEM, FEI Inc. Hillsboro, OR) was used to characterize the surface of coated graphite rod and transmission electron microscopy (TEM, 120 kV, JEM-1400, JOEL, Tokyo, Japan) was used to characterize the NPs on the annealed M-cPDA powder. In-situ X-ray diffraction (XRD, X’Pert Pro, Malvern PANalytical Ltd., Malvern, United Kingdom) was employed to conduct diffraction scans while heating up the powder samples. The temperature ranged from 50 to 850 °C with 5 °C steps. The XRD scan was conducted in the 2θ range between 40° and 50°, which contained the (111) peaks for Cu and Ni. Inductively coupled plasma mass spectrometry (ICP-MS, Agilent 7900, Agilent Technologies, Santa Clara, CA) was used to quantify the metal content in the powder samples.

### Catalytic performance study

Coated graphite rod samples were directly connected to the testing circuit and tested without further treatment after thermal annealing. However, only 15 mm of the rod was immersed under electrolyte to ensure consistent testing surface area. For comparison, powder samples were deposited on 3-mm diameter glassy carbon electrodes. Ten milligram of powder sample was mixed with 20 μL of Nafion™ binder, 5 mL water, and 5 mL alcohol with ultra-sonication. The mixed suspension was then drop-cast onto the electrodes using a pipette and dried in the oven. Commercially available Pt/C powder (Pt size ~ 3–4 nm, 40%, FuelCellStore, College Station, TX) was also tested in this study. M-cPDA powder fabricated by the previously mentioned method was used as a comparison to coated M-cPDA samples.

To evaluate the electrochemical behavior, current–voltage scans were conducted using an electrochemical workstation (Versastat 3, Ametek, Berwyn, PA) between 0 and −1 V with a scan rate of 0.005 V/s. Tests were conducted in sulfuric acid solution with a pH value of zero. Graphite rods and Ag/AgCl electrodes were used as the counter and the reference electrodes, respectively. To ensure the repeatability, at least three individual samples were tested for each type of materials.

## Results and discussions

**Figure 1:**
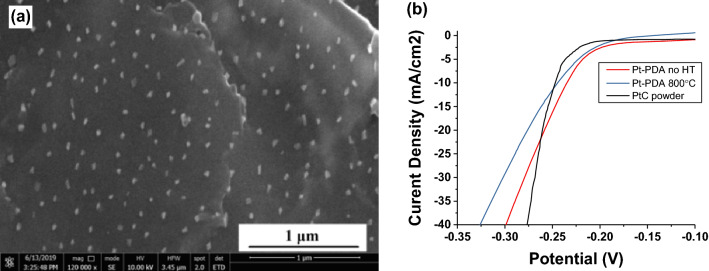
(**a**) SEM image of Pt-PDA coated on graphite rod; (**b**) catalytic performance of various samples (Pt-PDA, thermally annealed Pt-PDA, and commercial Pt/C) in pH = 0 sulfuric acid solution against Ag/AgCl electrode.

SEM analysis (Fig. 1a) showed that PtCl_4_ was successfully reduced to Pt NPs by the PDA thin film without heat treatment or addition of other reductants. Pt NPs decorated on the PDA film were uniform with an average diameter of around 20 nm while the commercial Pt/C powder contained 3–4 nm Pt nanoparticles. It is well known that the smaller the size of the active particles the better their performance is, as more surface area will be available for catalytic reactions. Therefore, reducing the particle size, even to single atoms, will enhance the Pt performance^22, 23^.

The HER performance of Pt-PDA against Ag/AgCl reference electrode is shown in Fig. 1b. All Pt-PDA samples showed lower overpotential at 10 mA/cm^2^ compared to the fabricated sample using commercial Pt/C powder. This may be attributed to the lesser thickness of the PDA film than the commercial Pt/C coating, which in turn resulted in low electrical resistance that is also important for catalytic performance. However, the Pt/C sample outperformed the Pt-PDA samples at higher current densities possibly because the smaller Pt particle size (3–4 nm) in the commercial paste than that obtained in our Pt-PDA sample (~ 20 nm). The overpotential of the Pt-PDA sample thermally annealed at 800 °C (245 mV at 10 mA/cm^2^) and was slightly higher than the sample without thermal treatment (236 mV at 10 mA/cm^2^). Although thermal annealing could reduce the electrical resistance of the PDA film through carbonization, it caused cracks in the PDA films due to thermal shrinkage which could lead to performance degradation.

Although Pt and other precious metal have outstanding performance as HER catalysts, their cost is a major concern in large-scale applications. In pursuit of less expensive alternatives with good HER efficiency, more abundant transition metal elements such as Ni, Cu, and their alloys have attracted much attention in recent years. For example, studies by Nady et al. had shown that the Ni-Cu alloy electroplated on Cu foil had better HER performance than their single element counterparts^24^ as alloying roughened the particle surface therefore increased the overall surface area of the metal cluster. On the other hand, our previous studies showed that transition metal nanocrystals could be fabricated by electron beam irradiation of metal-ion-containing PDA^25^. Therefore, the performance of Cu-PDA, Ni-PDA, and Cu–Ni-PDA as potential HER catalysts was evaluated in this study.

**Figure 2:**
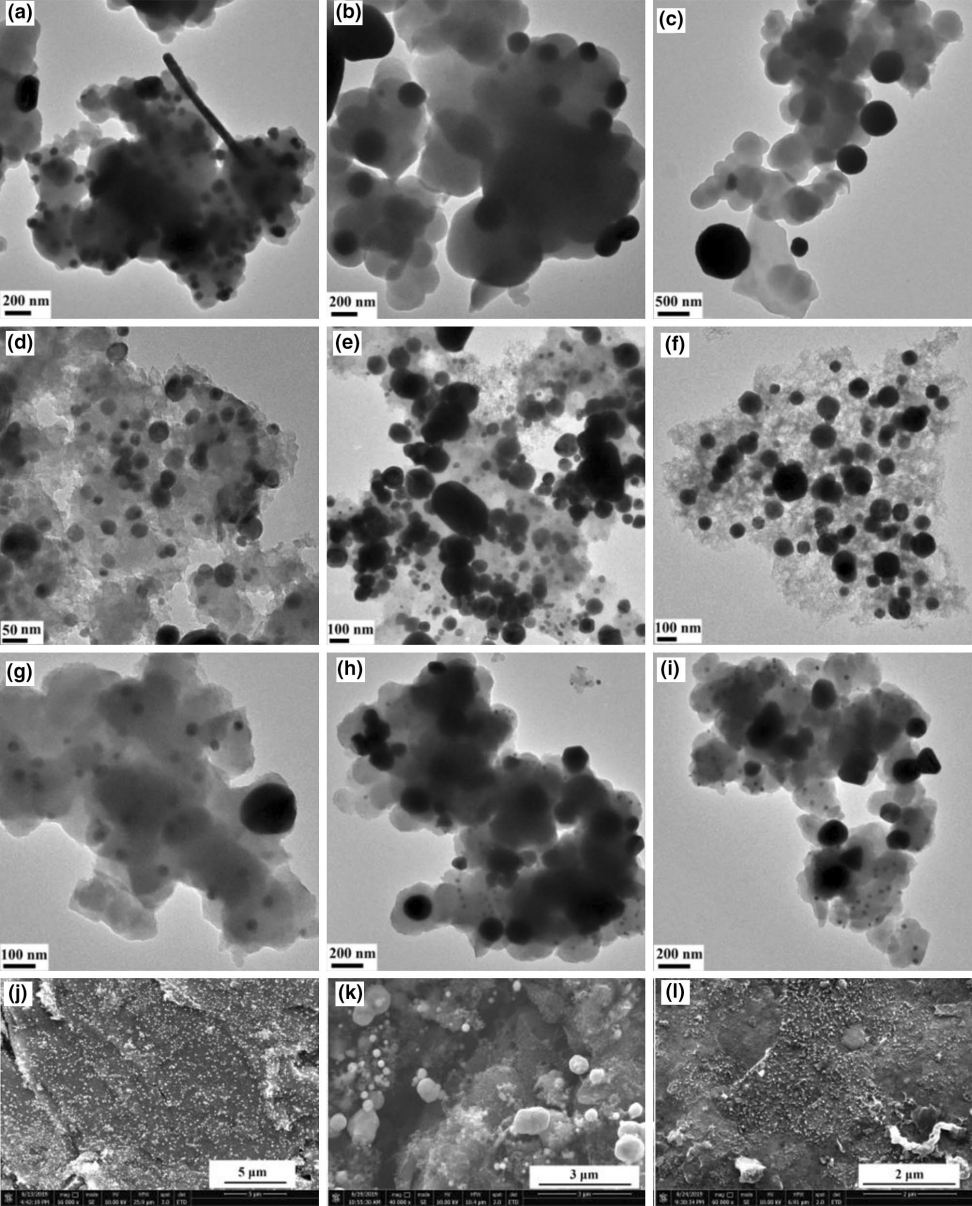
TEM images of Cu-PDA thermally annealed at (**a**) 600 °C, (**b**) 800 °C, (**c**) 1,000 °C; Ni-PDA thermally annealed at (**d**) 600 °C, (**e**) 800 °C, (**f**) 1,000 °C; and CuNi-PDA thermally annealed at (**g**) 600 °C, (**h**) 800 °C, (**i**) 1,000 °C. SEM images of M-PDA samples coated on graphite rods containing (**j**) Cu, (**k**) Ni, and (**l**) Cu–Ni NPs.

Cu, Ni, and Cu–Ni metal NPs were observed in powder samples after heat treatment at 600 °C and greater. As shown in Fig. 2, as annealing temperature increased, metal NPs appeared and merged into larger particles. This phenomenon was also observed in M-PDA samples from our previous study^15^. After heat treatment at 600 °C, the average size of Cu NPs was approximately 50 nm which increased to as large as 500 nm after heat treatment at 1,000 °C. In addition to equiaxed particles, nanorods were also observed in the Cu-PDA samples. In contrast, the size of Ni NPs was about 5 nm after heat treatment at 600 °C, which grew to approximately 100 nm after heat treatment at 1,000 °C. The CuNi-PDA samples showed a bimodal distribution of NPs with both large and small particles presented, which could be Cu and Ni NPs, respectively. Heat treatment at a higher temperature seemed to affect the stability of the metal NPs, such that the total number of NPs and their overall volume were reduced.

For M-PDA coated on graphite rods, Cu and Ni NPs were also observed. At 600 °C, Cu and Ni NPs were evenly distributed across the surface and it was similar to samples heat-treated at 800 °C (Fig. 2j, k). However, when heat-treated at 1,000 °C, Cu NPs merged into larger particles, which was similar to that observed on powder samples (Fig. 2c). Different from powder samples, bigger particles as large as 1 μm were observed on coated Ni-PDA film samples at 800 °C as shown in Fig. 2k. Furthermore, the increased intensity of the D and G peaks in the Raman spectroscopy profiles of M-PDA after the heat treatment clearly indicated the transition of PDA to carbonized PDA (Figure S4), which in turn enhanced the electrical conductivity^12^ and thus the overall catalytical performance.

Although many studies have focused on the structure of PDA^26, 27^, knowledge gaps still remain regarding the structure of metal-ion containing PDA materials. It has been shown that Cu^2+^ and Ni^2+^ were likely to form mono- or bis-catecholate with catechol groups^28^. In this study, ICP-MS was used to study the concentration of metal ions within the M-PDA system and determine M-PDA molar ratios. (Table 1). Assuming molar weight of each dopamine unit in PDA remains consistent, we could roughly calculate the molar ratio between metal ions to DA unit in M-PDA sample. For the Cu-PDA powder, Cu takes 6.5% of the total weight which roughly converted to a molar ratio of 1:6.11 for Cu to PDA. For the Ni-PDA powder, Ni takes 12% of the total weight and converted to a molar ratio of 1:2.78 for Ni to PDA. This result is similar to literature findings reported by Gianneschi’s group^29^, who found Cu weighs 8.0% (or 1:4.8 in terms of molar ratio) and Ni weighs 13.4% (or 1:2.5 in terms of molar ratio). Also, it is noteworthy that for the CuNi-PDA sample, Cu corresponded to 11.5% of the total weight with a metal-to-PDA ratio changed to 1:2.79, which is similar to the Ni:PDA ratio in the Ni-PDA sample (Table 1). In contrast, Ni only comprised 0.7% of the total weight in CuNi-PDA sample resulting a dramatic decrease in the Ni:PDA molar ratio of 1: 53.65. This implies that the majority Ni^2+^ ions were not incorporated in the PDA matrix. It is possible that when both Ni^2+^ and Cu^2+^ ions were present, Ni^2+^ first reacted with PDA to form Ni-PDA complex with an ion to PDA ratio of ~ 1:2.8. Then the Cu^2+^ ions substituted the Ni^2+^ with the same metal ion to PDA ratio. Literature findings report that when forming bio-complex coordination, Ni^2+^ exhibited higher activity while Cu^2+^ was more thermodynamically stable^30^, which could lead to the replacement of Ni^2+^ by Cu^2+^.

**Table 1:** ICP-MS results of metal to PDA molar ratio in M-PDA.

Sample	Cu-PDA	Ni-PDA	CuNi-PDA	
Metal type	Cu	Ni	Cu	Ni
Weight percentage (wt.%)	6.5	13.4	11.5	0.7
Metal to PDA ratio	1:6.11	1:2.78	1:2.79	1:53.65

**Figure 3:**
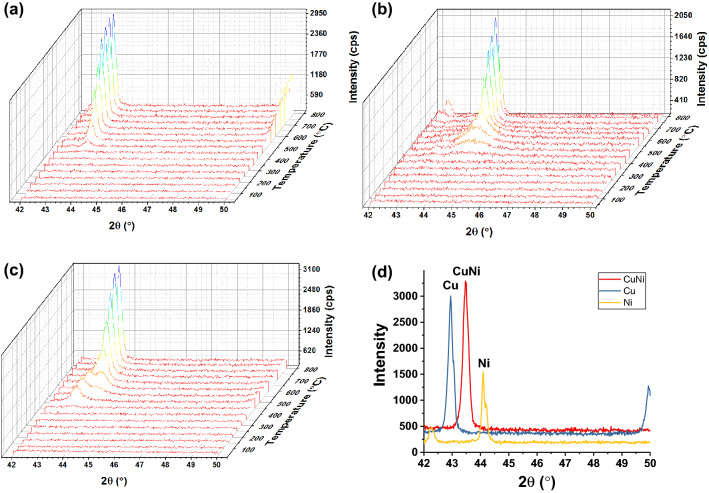
Temperature dependent in-situ XRD results for (**a**) Cu-PDA, (**b**) Ni-PDA, and (**c**) CuNi-PDA. (**d**) XRD peak of Cu, Ni, and CuNi-PDA annealed at 800 °C.

In-situ XRD was used to study the phase evolution in metal-PDA composites (Fig. 3). For Cu-PDA, the (111) peak at 2θ = 43° first appeared at 350 °C. For Ni-PDA, the (111) peak at 2θ = 44° first appeared at 380 °C. For the CuNi-PDA sample, a peak first appeared at 400 °C, which could be related to the precipitation of Cu. A second peak emerged at 450 °C, indicating the formation of Ni, which is believed to be an alloying phase between CuNi. As the temperature increased, the Cu peak at 43° decreased and the alloy peak at 43.5° increased. Above 600 °C, only the alloy peak could be observed^31^. This experiment showed that coordinatively bonded Cu and Ni ions in PDA could form alloys after thermal annealing.

**Figure 4:**
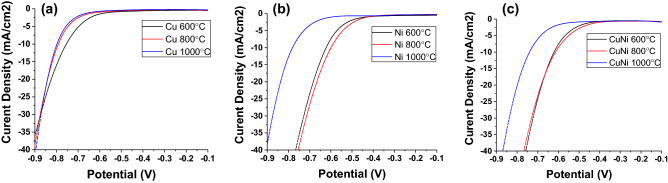
HER catalytic performance in pH = 0 sulfuric acid solution against the Ag/AgCl electrode. (**a**) Cu-PDA, (**b**) Ni-PDA, (**c**) CuNi-PDA. Samples were directly coated onto graphite rods and annealed at different temperatures.

Figure 4 shows the I–V curves of the metal-PDA samples against the Ag/AgCl reference electrode. For all types of samples, those thermally annealed at 600 °C had similar overpotential comparing to samples annealed 800 °C. However, significant increase in overpotential was observed in 1,000 °C samples. This could be due to the growth of metal NPs leading to reduced surface area (Fig. 2c, f, i). Cu-PDA on graphite annealed at 600 °C, 800 °C, and 1,000 °C with an overpotential of 555 mV, 592 mV, and 599 mV, respectively at a current density of 10 mA/cm^2^ (Fig. 4a). On the other hand, Ni-PDA showed a better performance of 423 mV, 400 mV, and 581 mV (Fig. 4b). Cu–Ni alloy sample yielded better performance than their single-element counterparts with overpotentials of 421 mV, 415 mV, 541 mV (Fig. 4c). The conductivity of carbonized PDA increases with annealing temperature^12^, and should result in a reduced overpotential, NP growth, and the reduction of surface area at higher temperature would lead to an increase in the overpotential. In addition to the growth of the NPs, the cracking in the PDA film induced by thermal shrinkage could also reduce its electrical conductivity and thus increase the overpotential. In comparison to the Pt/C and Pt-PDA samples included in this study (Fig. 1) and the literature data on Pd-PDA/CNH^21^, the Cu-PDA, Ni-PDA, and Cu–Ni-PDA showed higher overpotential at 10 mA/cm^2^, which may be attributed to the lower catalytic activities of the transition metals than the precious metals. Nevertheless, our results showed these transition metal nanoparticles supported on PDA films possessed certain catalytic activities, which could be further improved in the future.

## Conclusions

In this work, metal nanoparticles (NPs) were synthesized on polydopamine (PDA) supports. Platinum (Pt) was successfully reduced from Pt^4+^ by PDA without additional reducing agent. The resultant Pt NPs were evenly distributed on the PDA support and showed comparable catalytic performance to commercially used Pt/C in hydrogen evolution reaction. In addition, Cu, Ni, and Cu–Ni alloy NPs supported on PDA were synthesized with the assistance of thermal annealing and also demonstrated catalytic activities in HER. This work demonstrated the facile PDA synthesis strategy employing PDA could be used to synthesize metal NPs with catalytic activities. Furthermore, our results confirmed that PDA could be used as a platform to synthesize bimetallic alloys, such as Cu–Ni.

## Supporting information

Experimental details of SEM, TEM, ICP-MS, additional SEM, TEM images, and Raman spectrum can be found in supporting information.

## Supplementary information

Supplementary file 1

## References

[1] Lee H, Dellatore SM, Miller WM, Messersmith PB (2007). Mussel-inspired surface chemistry for multifunctional coatings. Science.

[2] Ball V (2018). Polydopamine films and particles with catalytic activity. Catal. Today.

[3] Im KM, Kim T-W, Jeon J-R (2017). Metal-chelation-assisted deposition of polydopamine on human hair: a ready-to-use eumelanin-based hair dyeing methodology. ACS Biomater. Sci. Eng..

[4] Chang T-L, Yu X, Liang JF (2018). Polydopamine-enabled surface coating with nano-metals. Surf. Coat. Technol..

[5] Tamakloe W, Agyeman DA, Park M, Yang J, Kang Y-M (2019). Polydopamine-induced surface functionalization of carbon nanofibers for Pd deposition enabling enhanced catalytic activity for the oxygen reduction and evolution reactions. J. Mater. Chem. A.

[6] Guo X, Zhang M, Zheng J, Xu J, Hayat T, Alharbi NS, Xi B, Xiong S (2017). Fabrication of Co@SiO_2_@C/Ni submicrorattles as highly efficient catalysts for 4-nitrophenol reduction. Dalton Trans.

[7] Liang Y, Wei J, Hu YX, Chen XF, Zhang J, Zhang XY, Jiang SP, Tao SW, Wang HT (2017). Metal-polydopamine frameworks and their transformation to hollow metal/N-doped carbon particles. Nanoscale.

[8] Liu C, Qiu Y, Xia Y, Wang F, Liu X, Sun X, Liang Q, Chen Z (2017). Noble-metal-free tungsten oxide/carbon (WOx/C) hybrid manowires for highly efficient hydrogen evolution. Nanotechnology.

[9] Son HY, Ryu JH, Lee H, Nam YS (2013). Silver-polydopamine hybrid coatings of electrospun poly(vinyl alcohol) nanofibers. Macromol. Mater. Eng..

[10] Du S, Luo Y, Liao Z, Zhang W, Li X, Liang T, Zuo F, Ding K (2018). New insights into the formation mechanism of gold nanoparticles using dopamine as a reducing agent. J Colloid Interface Sci.

[11] Zhao Y, Wu Z, Di Carlo F, Li H, Qian B, Feng Z, Ren F (2019). Enhancing the electrical and mechanical properties of copper by introducing nanocarbon derived from polydopamine coating. J. Alloy. Compd..

[12] Li H, Aulin YV, Frazer L, Borguet E, Kakodkar R, Feser J, Chen Y, An K, Dikin DA, Ren F (2017). Structure evolution and thermoelectric properties of carbonized polydopamine thin films. ACS Appl. Mater. Interfaces..

[13] Kong J, Yee WA, Yang L, Wei Y, Phua SL, Ong HG, Ang JM, Li X, Lu X (2012). Highly electrically conductive layered carbon derived from polydopamine and its functions in SnO_2_-based lithium ion battery anodes. Chem. Commun..

[14] Li R, Parvez K, Hinkel F, Feng X, Mullen K (2013). Bioinspired wafer-scale production of highly stretchable carbon films for transparent conductive electrodes. Angew. Chem..

[15] Li H, Marshall T, Aulin YV, Thenuwara AC, Zhao Y, Borguet E, Strongin DR, Ren F (2019). Structural evolution and electrical properties of metal ion-containing polydopamine. J. Mater. Sci..

[16] Li J, Zheng G (2017). One-dimensional earth-abundant nanomaterials for water-splitting electrocatalysts. Adv Sci (Weinh).

[17] Du Z, Wang Y, Li J, Liu J (2019). Facile fabrication of Pt–Ni alloy nanoparticles supported on reduced graphene oxide as excellent electrocatalysts for hydrogen evolution reaction in alkaline environment. J. Nanoparticle Res..

[18] Greeley J, Jaramillo TF, Bonde J, Chorkendorff IB, Norskov JK (2006). Computational high-throughput screening of electrocatalytic materials for hydrogen evolution. Nat Mater.

[19] Qu K, Zheng Y, Dai S, Qiao S (2015). Polydopamine-graphene oxide deried mesoporous arbon nanosheets for enhanced oxygen reduction. Nanoscale.

[20] Qu K, Wang Y, Vasileff A, Jiao Y, Chen H, Zheng Y (2018). Polydopamine-inspired nanomaterial for energy conversion and storage. J. Mater. Chem. A..

[21] Devadas B, Chang C, Imae T (2019). Hydorgen evolution reation efficiency of carbon nanohorn incorporating molybdenum sulfide and polydopamine/palladium nanoparticles. J. Taiwan Inst. Chem. Eng..

[22] Luo W, Gan J, Huang Z, Chen W, Qian G, Zhuo X, Duan X (2019). Boosting HER performance of Pt-based catalysts immobilized on functionalized vulcan carbon by atomic layer deposition. Front. Mater..

[23] Cheng N, Stambula S, Wang D, Bains MN, Liu J, Riese A, Xiao B, Li R, Sham T, Liu L, Botton GA, Sun X (2016). Platinum single-atom and cluster catalysis of the hydrogen evolution reaction. Nat Commun.

[24] Nady H, Negem M (2016). Ni–Cu naon-crystalline alloys for efficient electrochemical hydrogen prodution in acid water. RSC Adv..

[25] Li H, Zhao Y, Zhang Z, Andaluri G, Ren F (2019). Electron-beam induced *in situ* growth of self-supported metal nanoparticles in ion-containing polydopamine. Mater. Lett..

[26] Dreyer DR, Miller DJ, Freeman BD, Paul DR, Bielawski CW (2013). Perspectives on poly(dopamine). Chem. Sci..

[27] Delparastan P, Malollari KG, Lee H, Messersmith PB (2019). Direct evidence for the polymeric nature of polydopamine. Angew. Chem..

[28] Sever MJ, Wilker JJ (2004). Visible absorption spectra of metal–catecholate and metal–tironate complexes. Dalton Trans..

[29] Wang Z, Xie Y, Li Y, Huang Y, Parent LR, Ditri T, Zang N, Rinehart JD, Gianneschi NC (2017). Tunable, metal-loaded polydopamine nanoparticles analyzed by magnetometry. Chem. Mater..

[30] Brewer DG, Wong PTT, Sears MC (1968). The nature of the coordination bond in metal complexes of substituted pyridine derivatives. III. 4-metylpyridine complexes of some divalent transition metal ions. Can. J. Chem..

[31] Zhang Y, Zuo TT, Tang Z, Gao MC, Karin A, Dahmen PK,  Liaw PK,  Liaw PK, Lu  ZP (2014). Microstructures and properties of high-entropy alloys. Prog. Mater. Sci..

