# Supplementary material for: Synthesis and catalytic performance of polydopamine supported metal nanoparticles
Source: Sci Rep. 2020 Jun 26;10:10416. doi: 10.1038/s41598-020-67458-9 (PMC7319955; doi:10.1038/s41598-020-67458-9)
Supplement: Supplementary file 1 [file 41598_2020_67458_MOESM1_ESM.docx]

Synthesis and catalytic performance of polydopamine supported metal nanoparticles

Haoqi Li^1^, Jiaxin Xi^1^, Adrienne Donaghue^2^, Jong Keum^3^, Ke An^3^, Yao Zhao^1^, Erica McKenzie^2^, Fei Ren^1^

^1^Department of Mechanical Engineering, Temple University, Philadelphia, PA 19122, USA

^2^Department of Mechanical Engineering, Temple University, Philadelphia, PA 19122, USA

^3^Chemical and Engineering Materials Division, Oak Ridge National Laboratory, Oak Ridge, TN 37831, USA

Supplementary information

**Experimental methods**

SEM

The heat-treated M-cPDA coated graphite rod are directly placed on SEM sample stage with adhesive carbon tape. The acceleration voltage is 10kV and spot size of 2.

TEM

Collected M-PDA powder are dispersed in isopropanol alcohol and ultrasonicated for 5min. One drop (20μL) of the mixed suspension is collected by pipette and cast on copper TEM grids. Casted grid is then dried in the oven at 50 ˚C overnight.

ICP-MS

On average, 1.6mg of dry powder sample was dispersed in 10mL in acidified sub-boiled water (2% nitric and 0.5% hydrochloric acid). Digested M-PDA samples were prepared in duplicated and sonicated for a minimum of 15 minutes to ensure homogenization. Following sonication, the suspension was diluted for ICP-MS analysis and measured in triplicate.

Raman spectroscopy

To characterize the carbon phase in the metal NPs decorated, cPDA thin film, Raman spectroscopy was used with a 532 nm green laser for excitation.

**Additional SEM and TEM images**

Pt-PDA annealed at 800 ˚C (Figure S1) showed the coating shrinkage and cracks on the surface. The Pt NP size and spatial distribution are also disturbed due to annealing.


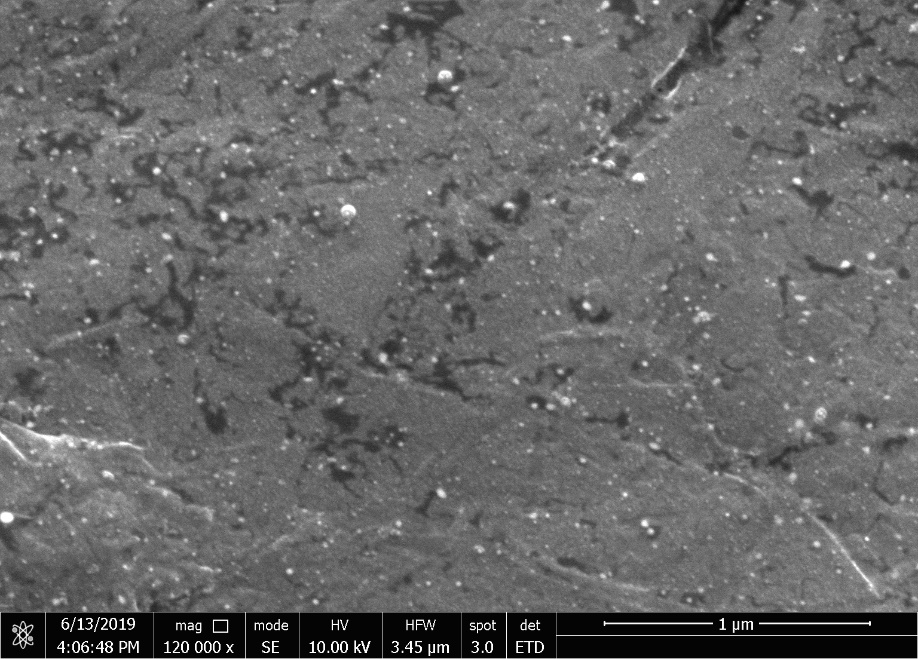


Figure S1: Pt-PDA coated on graphite rod after 800 ˚C annealing.

The size of Ni Nanoparticles is very small (~5nm) at the early stage of their growth as shown in figure S2. They grow larger as annealing temperature increases.





Figure S2: TEM image of Ni-PDA 400

Figure S3 showed a SEM image of Cu rod grow from Cu-PDA coating. The longest rod found is 14μm.


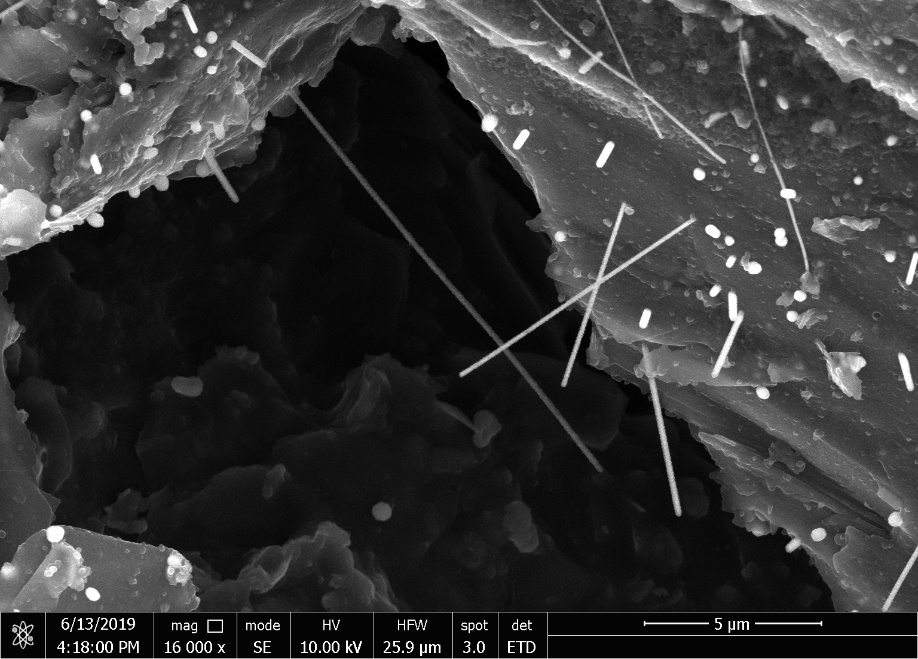


Figure S3: Cu-PDA annealed at 600 ˚C showed Cu rod formed on the surface.

*Raman spectroscopy*

Raman spectrum of all M-PDA coating before and after heat treatment has similar results. Typical spectrums are shown in figure S4. The peak around 800 cm^-1^ came from Si/SiO_2_ substrate and could be used as a reference. There are other two significant peaks at 1350 and 1600 cm^-1^ corresponds to D and G peak of sp^2^ carbon. Comparing figure S4a and S4b, D and G peak undergoes a significant enhancement, representing the carbonization of PDA and conversion of amorphous PDA to nanocrystal carbon.

Figure S4: typical Raman spectrum of M-cPDA thin film (a) before and (b) after annealing.
